# Chronic, Episodic Nicotine Alters Hypoglossal Motor Neuron Function at a Critical Developmental Time Point in Neonatal Rats

**DOI:** 10.1523/ENEURO.0203-21.2021

**Published:** 2021-08-12

**Authors:** Lila Buls Wollman, Ralph F. Fregosi

**Affiliations:** 1 Department of Physiology, The University of Arizona, Tucson, AZ 85724; 2 Department of Neuroscience, The University of Arizona, Tucson, AZ 85724

**Keywords:** firing rate, homeostatic plasticity, neuron development, neurotoxin, pattern sensitivity, potassium conductance

## Abstract

Developmental nicotine exposure (DNE), alters brainstem neurons that control breathing, including hypoglossal motor neurons (XIIMNs), which innervate the tongue. Here, we tested the hypothesis that chronic, episodic DNE (eDNE), which mimics nicotine replacement therapies such as e-cigarettes or nicotine gum, alters the function of nicotinic acetylcholine receptors (nAChRs), XIIMN intrinsic properties, and tongue muscle function *in vivo* similar to what we have observed with a chronic, sustained exposure model. We delivered nicotine to pregnant Sprague Dawley rats through drinking water and studied pups of either sex in two age groups: postnatal day (P)1–P5 and P10–P12, which encompasses a critical period in brain development. At P1–P5, eDNE was associated with delayed recovery of nAChRs from desensitization; however, there were no changes in the magnitude of desensitization, XIIMN intrinsic properties, or tongue muscle function *in vivo.* By P10–P12, eDNE XIIMNs had lower peak firing frequencies in response to depolarizing current injection, larger delayed rectifier potassium currents, and continued to exhibit delayed nAChR recovery. Moreover, this age group exhibited a blunted and delayed tongue muscle response to nasal occlusion *in vivo*, indicating that changes to XIIMN intrinsic properties is an important mechanism behind this effect, as it is not produced by altered nAChR function alone. Together, these results show that eDNE alters XIIMNs and tongue muscle function during a critical period in brain development and that the specific effects of chronic nicotine exposure may be pattern dependent.

## Significance Statement

Disruption of nicotinic cholinergic signaling, as occurs with perinatal nicotine exposure, alters the development of the neural respiratory network and is strongly associated with sudden infant death syndrome (SIDS). The specific dose, time between doses, and route of administration of nicotine determine its specific developmental effects. Here, we show that chronic, episodic perinatal nicotine exposure alters nicotinic acetylcholine receptor (nAChR) function, hypoglossal motor neuron (XIIMN) intrinsic properties, and respiratory-related tongue muscle function *in vivo.* These findings, while similar to what has been shown using a previous model studying chronic, sustained nicotine exposure, are unique in that they are prominent at a critical period in brain development in rodents and reveal important mechanisms that further link perinatal nicotine exposure to altered brain development and the pathophysiology of SIDS.

## Introduction

Brain development from embryonic stages through adolescence is dependent on the trophic effects of acetylcholine (ACh) acting on the nicotinic ACh receptors (nAChRs; Greer et al., 2006; Dwyer et al., 2008). Disruption of nicotinic signaling alters brain morphology and function, leading to abnormalities in brain physiology including in brainstem regions that control breathing. This has been extensively studied in the context of perinatal exposure to nicotine, a competitive agonist of the nAChR. Infants and children born to mothers that smoked during and after pregnancy have abnormalities in hypoglossal motor neurons (XIIMNs), the neurons that innervate the tongue muscles, and increased incidence of obstructive apneas and other alterations to cardiorespiratory control that are considered potential mechanisms underlying sudden infant death syndrome (SIDS; Sawnani et al., 2004; Bednarczuk et al., 2020).

In studies using rodent models, developmental nicotine exposure (DNE; prenatal nicotine exposure with continued exposure after birth) causes various changes to XIIMNs and tongue muscle control, including altered nAChR function, intrinsic membrane properties of XIIMNs and breathing-related tongue muscle function *in vivo* (Robinson et al., 2002; Pilarski et al., 2011, 2012; Cholanian et al., 2017; Wollman et al., 2019). In the majority of prior work, the osmotic minipump method was used for nicotine delivery, which exposes pups to nicotine continuously, owing to a constant infusion of nicotine delivered to a pregnant dam, and mimics the use of the nicotine patch. However, nicotine exposure through cigarettes or nicotine delivery devices such as e-cigarettes or gum is episodic. Limited randomized control trials concluded that episodic nicotine replacement therapies such as these, while controversial, are safer during pregnancy than continuous use formulations because they reduce the total dose of nicotine delivered to the fetus (Sailer et al., 2019). However, in animal studies, even brief periods of nicotine exposure can trigger neuroplasticity that has long-term developmental consequences (Abreu-Villaça et al., 2003). Moreover, in many systems, the specific expression of plasticity is dependent on the time course or periodicity of a given treatment. For example, in the respiratory network, intermittent hypoxia reliably elicits a sustained increase in respiratory motor output, which is not observed with the same duration of sustained hypoxia, indicating that the mechanism behind this form of respiratory plasticity is pattern dependent (Baker and Mitchell, 2000; Mitchell et al., 2001). With the present study, we test the effects of chronic, episodic DNE (eDNE) on the development of XIIMNs and tongue muscle control *in vivo.* In addition, although previous work offers information about the influence of nicotine exposure on the function of XIIMNs in rats within the first week of life, the persistence of these effects is unknown. In rats, the respiratory system is advanced enough at birth to produce gas exchange and coordinate critical behaviors such as swallowing (Greer et al., 2006). However, significant postnatal maturation occurs during the first few postnatal weeks, including growth and refinement of inhibitory synapses during a “critical period” near the end of the second postnatal week (Wong-Riley et al., 2019). Therefore, we will study neonatal rats at postnatal day (P)1–P5 and also at P10–P12, which encompasses days leading up to, and within this critical window in respiratory system development (Mu et al., 2018; Wong-Riley et al., 2019).

We exposed pregnant dams to nicotine through drinking water and designed experiments to test the hypothesis that eDNE alters XIIMN intrinsic properties and nAChR desensitization kinetics *in vitro*, and tongue muscle electromyographic (EMG) activity *in vivo*. We found that eDNE was associated with nAChR desensitization at P1–P5, but did not alter XIIMN firing properties, whole-cell potassium currents, or tongue muscle EMG activity. However, by P10–P12, XIIMNs from eDNE pups had blunted action potential firing rates, accompanied by larger potassium currents compared with neurons from control pups. This was associated with blunted tongue muscle EMG responses to nasal occlusion. Together, these results suggest that the pattern of nicotine exposure is an important regulator of developmental plasticity in XIIMNs, and that chronic, eDNE results in altered XIIMN properties and tongue muscle function at a critical period in respiratory system development.

## Materials and Methods

### Animals

Pups were born via spontaneous vaginal delivery from pregnant adult female Sprague Dawley rats purchased form Charles River Laboratories. Pups were housed with their mothers and littermates in the animal care facility (12/12 h light/dark cycle, 22°C, 30–70% relative humidity) with food and water available *ad libitum.* For all experiments we used rat pups of either sex within two age groups: (1) P1 through P5 and (2) P10 through P12, which approximately correspond with 23–35 weeks (preterm infant) and 40 weeks (full term infant) in humans. P10–P12 represents the days just before, and encompassing a critical period for the development of brainstem neurons in rats (Semple et al., 2013). All procedures and protocols were approved by the Institutional Animal Care and Use Committee and are in accordance with National Institutes of Health Guidelines.

### eDNE

Nicotine-exposed pups were obtained from pregnant dams who were exposed to chronic, episodic nicotine through drinking water starting on embryonic day (E)5. This results in fetal exposure by the placental circulation *in utero,* and through breast milk after birth. Nicotine at a concentration of 0.08 mg/ml of tap water was mixed with 10 mg/ml of saccharin for palatability. The nicotine-saccharin water mixture was prepared fresh and replaced every 3 d. This dose of nicotine produced plasma cotinine (a stable metabolic by-product of nicotine) levels in the pups ranging from ∼50–100 ng/ml; however, there were samples at each age where cotinine levels were higher than the measurable limit of the assay (Cotinine ELISA, CalBiotech). This range of plasma cotinine levels is similar to what we have previously observed in neonates exposed to chronic, sustained nicotine via an osmotic minipump set to deliver a nicotine dose of 6 mg/kg/d (60–92 ng cotinine/ml plasma; Powell et al., 2015). These cotinine levels are also comparable to what is found in the plasma of human infants born to mothers who are considered moderate smokers (Berlin et al., 2010). Control pups were obtained from pregnant dams who were given saccharin water only.

### Medullary slice preparations

#### P1–P5

Procedures were followed as described previously (Wollman et al., 2018b,c, 2019). Pups of either sex were removed from their cages, weighed, anesthetized on ice and decerebrated at the coronal suture. The vertebral column and ribcage were exposed and placed in cold (4–8°C) oxygenated (95% O_2_–5% CO_2_) artificial CSF (aCSF), composed of the following: 120 mM NaCl, 26 mM NaHCO_3_, 30 mM glucose, 1 mM MgSO_4_, 3 mM KCl, 1.25 mM NaH_2_PO_4_, and 1.2 mM CaCl_2_ with pH adjusted to 7.4 and osmolarity to 300–325 mOsm. The brainstem, and spinal cord were extracted and all tissue above the pontomedullary junction was removed. The preparation was then glued to an agar block, rostral surface up, and two to three transverse medullary slices (250 μm thick) containing the hypoglossal motor nucleus were cut in a vibratome (VT 1200, Leica) filled with ice-cold, oxygenated aCSF. The slices were then transferred to an equilibration chamber containing fresh, oxygenated, room temperature aCSF and allowed to recover for 1 h before recording.

#### P10–P12

Procedures for tissue preparation were followed as described by Ting et al. (2014), to reduce damage to the superficial layers of the slice and improve the success rate for patch clamp recordings in older rodents (Ting et al., 2014). Pups of either sex were removed from their cages, weighed, deeply anesthetized with 5% isoflurane in room air, and then transcardially perfused with ice-cold, oxygenated NMDG aCSF composed of the following: 92 mM NMDG, 2.5 mM KCl, 1.25 mM NaH_2_PO_4_, 30 mM NaHCO_3_, 20 mM HEPES, 25 mM glucose, 2 mM thiourea, 5 mM Na-ascorbate, 3 mM Na-pyruvate, 0.5 mM CaCl_2_·4H_2_O, and 10 mM MgSO_4_·7H_2_O with pH adjusted to 7.3. After perfusion, the brainstem and spinal cord were prepared as above for slicing in a vibratome filled with ice-cold, oxygenated NMDG aCSF. Slices were then transferred to a recovery chamber containing oxygenated NMDG aCSF maintained at 32°C for 12 min. After this initial recovery period, slices were transferred into a new holding chamber containing room-temperature, oxygenated HEPES holding aCSF composed of the following: 92 mM NaCl, 2.5 mM KCl, 1.25 mM NaH_2_PO_4_, 30 mM NaHCO_3_, 20 mM HEPES, 25 mM glucose, 2 mM thiourea, 5 mM Na-ascorbate, 3 mM Na-pyruvate, 2 mM CaCl_2_·4H_2_O, and 2 mM MgSO_4_·7H_2_O with pH adjusted to 7.3.

### *In vitro* electrophysiology

Equilibrated slices were transferred to a recording chamber and perfused with aCSF (see P1–P5 slicing methods above) at a rate of 1.5 ml/min. aCSF was oxygenated and maintained at 27°C (TC-324B temperature controller, Warner Instrument Corporation). XIIMNs were visualized with an Olympus BX-50WI fixed-stage microscope (40× water-immersion objective, 0.75 N.A.) with differential contrast optics and a video camera (C2741-62, Hamamatsu). Whole cell patch clamp recordings were made with glass pipettes (tip resistance 3–7 MΩ) pulled from thick-walled borosilicate glass capillary tubes (OD: 1.5 mm, ID: 0.75 mm) filled with intracellular solution containing the following: 135 mm K-gluconate, 4 mm KCl, 12.5 mm disodium phosphocreatine, 10 mm HEPES, 0.375 mm Na-GTP, and 5 mm ATP (Mg^2+^ salt) with pH adjusted to 7.3 and osmolarity adjusted to 250–275 mOsm. Pipettes were attached to a head stage mounted in a micromanipulator (MP-225, Sutter Instrument Company) connected to a Multiclamp 700B amplifier, and signals were digitized with a Digidata 1440A A/D converter (Molecular Devices). Current and voltage signals were amplified and acquired using the Axoclamp 1D system, pClamp software, and a Digidata 1320 AD/DA data acquisition system (Molecular Devices). Signals were low-pass filtered at 1.0 kHz, digitized at 20 kHz, observed during the experiment in real time, and stored on a computer hard drive (Gateway).

### Protocols and drugs

XIIMNs were identified visually based on their size and location and approached in voltage clamp mode. After junction potentials were zeroed and a gigaohm seal was achieved, the membrane was ruptured by suction. Cells were initially held in voltage clamp mode at −70 mV for a 5-min equilibration period to confirm a stable recording. XIIMNs were then assessed in current clamp, and cells with a resting membrane potential more positive than −40 mV and/or with action potential amplitudes that did not exceed 0 mV were not studied.

#### Experimental set 1, XIIMN firing properties

To examine the effects of eDNE on the action potential firing rate in response to injected current, we used a current step protocol consisting of a series of 250 ms duration current pulses ranging from −100 to +1000 pA in 50-pA increments. Although we did not use a current ramp test to precisely measure the rheobase current and the current associated with depolarization block, we did note and compare the current step at which each neuron initiated and ceased action potential firing. After the current step protocol, we switched back to voltage clamp gap free mode and recorded the holding current at −70 mV. For the following experiments, the slow and fast components of the capacitive current transient were cancelled to the extent possible, and series resistance was compensated with a correction coefficient of 60%. After these corrections were made, a square wave voltage step from −70 to −80 mV was introduced to calculate the input resistance of the neuron.

#### Experimental set 2, potassium currents

Here, we observed that XIIMNs from control and eDNE pups respond differently to depolarizing current injection (see Results). Therefore, we next ran three separate voltage-step protocols to study voltage-gated potassium currents that contribute to repetitive action potential firing properties in XIIMNs, as described in detail previously (Bayliss et al., 1994; Cholanian et al., 2017; Thakre and Bellingham, 2019). Before these experiments the superfusate was switched to aCSF containing the voltage-gated sodium channel blocker tetrodotoxin (TTX; a, 1 μm, Sigma). After 5 min of equilibration in TTX, we recorded the transient and sustained components of the whole-cell potassium current with a voltage-step protocol consisting of a series of 400-ms duration voltage pulses ranging from −100 to +10 mV in 10-mV increments.

The voltage-gated, rapidly inactivating A-type potassium current is activated near spiking threshold and plays an important role in time and voltage-dependent regulation of action potential firing (Fransén and Tigerholm, 2010). To estimate the contribution of the A-type potassium current to the transient component of the whole-cell current trace, we next ran a protocol that consisted of a “prepulse” voltage step. From a holding potential of −70 mV, we stepped the membrane potential to −30 mV for 35 ms to inactivate the A-current, and this was followed by twelve, 200-ms duration square-wave pulses through the voltage range described above.

We then used the following protocol to isolate the voltage dependent, delayed rectifier current. This current, which activates slowly and shows little time-dependent inactivation, influences action potential repolarization and spike frequency (Liu and Bean, 2014). From a holding potential to −40 mV, we delivered a series of 200-ms depolarizing pulses in 10-mV increments to a final value of +40 mV.

#### Experimental set 5, nAChR desensitization kinetics

In a final *in vitro* experiment, we assessed the function of nAChRs located postsynaptically on XIIMNs as described previously (Pilarski et al., 2012). In the presence of TTX, neurons were held at a resting potential of −70 mV and we used pressure pulses (Picospritzer II) to deliver nicotine (2 mm, Sigma), an nAChR agonist, to test nAChR desensitization and recovery. Nicotine was dissolved in aCSF, loaded into a patch pipette, and positioned near the XIIMN of interest. Nicotine was pressure injected onto the XIIMN at predetermined interpulse intervals and the resulting inward currents recorded. Pressure pulses of nicotine were delivered at 5, 30, 60, 120, and 240 s after a control (baseline) pulse, resulting in increasingly longer interpulse intervals (5, 25, 30, 60, and 120 s). Neurons that did not produce a measurable current response to all six current pulses were excluded. Although nicotine concentration remained constant for all experiments, pressure (12–25 psi), pulse duration (10–20 ms), and distance from the soma (20–60 μm) were optimized varied depending on the age to give a strong initial response that did not disrupt the integrity of the patch, but also ensured a measurable response to the second pulse, where most receptors were desensitized.

### EMG recordings of tongue and diaphragm muscles

Rat pups were lightly anesthetized with a mixture of ketamine (30 mg/ml), xylazine (6 mg/ml), and acepromazine (3 mg/ml), injected subcutaneously at a volume corresponding to ∼0.35 μl/g body weight. Pain sensitivity was assessed via multiple paw pinches initiated 10 min after time of anesthetic injection. Supplemental anesthetic was added until paw retraction to pinch was abolished. With the pup in the supine position, fine wire electrodes were used to record whole muscle EMG activity of the diaphragm muscle and the genioglossus (GG) (a tongue protrudor muscle) as described previously (Janssen et al., 2000; Bailey et al., 2001, 2005; Rice et al., 2011; Wollman et al., 2019). An additional electrode placed into the scruff of the neck served as an electrical ground. EMG signals were filtered (30–3000 Hz), amplified (Grass P122 AC amplifiers), and sent to an A/D converter [Cambridge Electronic Design (CED); model 1401], which sampled the signal at a rate of 8300 Hz. EMG signals were displayed in real time on a computer monitor (Spike2 software) and stored on a hard drive for analysis offline.

After implantation of EMG electrodes, a stable baseline was recorded for at least 10 min, after which the animal was challenged with nasal occlusions resulting in strong breathing efforts but an absence of lung inflation, as well as hypoxia, hypercapnia, and acidosis. Each animal was subjected to three 15-s nasal occlusions, with 5 min of rest in between measurements of peak GG EMG activity were taken in 2-min bins throughout each nasal occlusion period and averaged across the three occlusion challenges. After the experiment, animals were deeply anesthetized with isoflurane and decapitated.

### Analysis and statistics

For both *in vitro* electrophysiology and *in vivo* EMG experiments, animal weight and age were compared between control and eDNE across ages using a two-way ANOVA with group means compared using Tukey’s *post hoc* test (P1–P5: data obtained from *n* = 8 control and *n* = 8 eDNE pups; P10–P12: data obtained from *n* = 9 control and *n* = 8 eDNE pups). For initial analyses, animals within each group (i.e., control and eDNE) at each age (i.e., P1–P5 and P10–P12) were compared for sex differences. Consistent with previous work, we did not find any differences between males and females within treatment groups for any of the parameters measured, thus, we combined males and females for the analyses below.

#### *In vitro* electrophysiology

All *in vitro* electrophysiology data were analyzed with Clampfit (Molecular Devices). Input resistance was calculated by measuring the current response evoked by a rapid 10-mV hyperpolarizing step from a holding potential of −70 mV. Resting membrane potential, holding current, input resistance, estimated firing threshold, and the current corresponding with depolarization block were compared between neurons from control and eDNE animals across ages using a two-way ANOVA with group means compared using Tukey’s *post hoc* test (data obtained from *n* = 16 neurons in each group at each age). The relationship between current injection and action potential frequency was compared between control and eDNE at each age using a two-way, mixed-model repeated measures ANOVA, with treatment (control vs eDNE) and time as main factors (data obtained from *n* = 12 control and eDNE neurons in each age group).

For voltage-step experiments, leak current was subtracted offline and all current amplitudes were obtained from the leak subtracted traces. The transient component of the whole-cell potassium current was measured immediately after the capacitive artifact, while the sustained current component was measured at the end of the voltage steps. Delayed rectifier currents were measured at the end of the 200-ms voltage steps. Voltage-current relationships were compared at each age using a two-way, mixed-model repeated measures ANOVA, with treatment (control vs eDNE) and time as the main factors (data obtained from *n* = 12 control and eDNE neurons in each age group).

Desensitization and recovery of nAChRs was quantified first by calculating the peaks of each current response to nicotine pressure injection, and then normalizing the second through sixth current peaks to the peak of the first (baseline) current response. Average amplitudes were compared at each age using a two-way, mixed-model repeated measures ANOVA, with treatment (control vs eDNE) and time as the main factors, and Sidak’s *post hoc* test to assess individual significant differences. Recovery time course was calculated as the time constant (τ) of a single parameter exponential equation (P1–P5: data obtained from *n* = 10 control and eDNE neurons; P10–P12: data obtained from *n* = 5 control and eDNE neurons).

#### *In vivo* EMG

The time between the onset of nasal occlusion and the first GG EMG burst during nasal occlusion was defined as the response latency, and differences between control and eDNE across ages were compared using a two-way ANOVA with group means compared using Tukey’s *post hoc* test. The average GG EMG burst amplitude within each challenge was normalized to the maximal burst amplitude achieved. Changes in GG EMG activity during nasal occlusion were compared at each age using a two-way, mixed-model repeated measures ANOVA, with treatment (control vs eDNE) and time as main factors, and Sidak’s *post hoc* test to assess individual significant differences (data obtained from eight control and eight eDNE pups in each age group).

All statistical analyses were performed using Prism 8 (GraphPad). For all analyses, *p* < 0.05 was used as the threshold for statistical significance. All experimental data are presented as mean ± SD.

## Results

### Weight and age of pups, and resting membrane potential, input resistance, and holding current of XIIMNs

**Figure 1.**
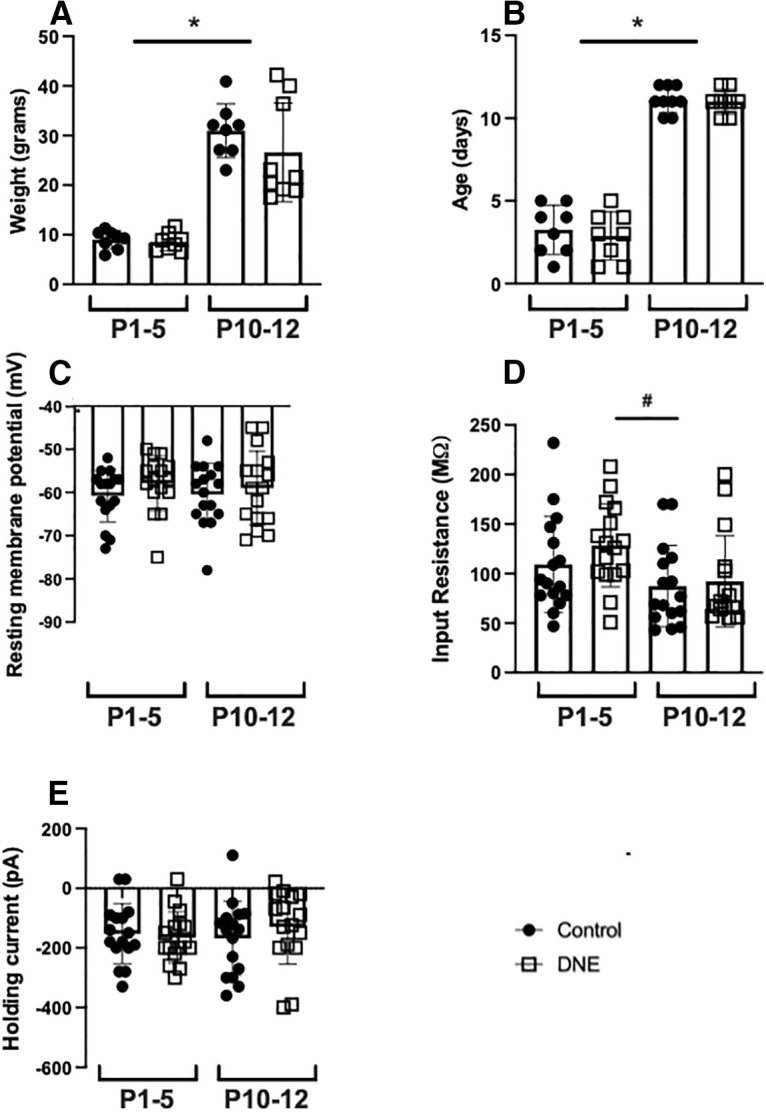
Weight, age, resting membrane potential, input resistance, and holding current. There were no differences in weight (***A***) or age (***B***) between control (closed circles) and eDNE (open squares) pups at either age (P1–P5: *n* = 8 control and *n* = 6 eDNE; P10–P12: *n* = 11 control and *n* = 8 eDNE). There was also no difference in resting membrane potential (***C***), input resistance (***D***), or holding current (***E***) of XIIMNs from control (closed circles) and eDNE (open squares) pups at either age (*n* =* *16 control and *n* =* *16 eDNE neurons in each age group). All data presented as mean ± SD; * indicates significant difference between age groups. #, significant difference between P1-P5 eDNE preparations and P10-P12 control preparations.

As shown in Figure 1, there was no difference in the weight (*p* = 0.2652; Fig. 1*A*) or age (*p* = 0.9352; Fig. 1*B*) of control and eDNE pups within each of the age groups. As expected, animals in the P10–P12 age group weighed significantly more than animals in the P1–P5 age group (P1–P5: *n* = 8 control and eDNE pups; P10–P12: *n* = 9 control and *n* = 8 eDNE pups). A total of 64 neurons from 33 pups across 19 litters (10 control and 9 eDNE) were used for these experiments. Resting membrane potential (*p* = 0.6435; Fig. 1*C*) and holding current (*p* = 0.0578; Fig. 1*E*) did not differ between XIIMNs from control or eDNE pups at either age, nor did they differ between age groups. There was no difference in input resistance between control and eDNE neurons within each age group, and input resistance did not change with age in either treatment group. eDNE neurons in the P1–P5 age group, however, had a significantly higher average input resistance than control neurons at the P10–P12 age group (*p* = 0.0409; Fig. 1*D*; data from *n* = 16 control and eDNE neurons in each age group). These data are summarized in Table 1. An additional analysis of input resistance was performed for the neurons used to collect potassium current data (see below). Input resistance measures in this subset of neurons were not different between control and eDNE at either age (*n* = 12 control and eDNE neurons in each age group; data not shown).

**Table 1.** Average data for weight, age resting membrane potential (RMP), holding current (I_Hold_), and input resistance (R_IN_) in control and eDNE groups in each of the two age groups

	Control	eDNE	*n* (control:eDNE)	*p* value
Weight (g)				
P1–P5	9.01 ± 0.6	8.6 ± 0.6	8:8 pups	<0.0001
P10–P12	**26.59 ± 3.3**	**31.0 ± 1.9**	9:8 pups	
Age (d)				
P1–P5	3.25 ± 0.5	2.88 ± 0.5	8:8 pups	<0.0001
P10–P12	**11.11 ± 0.3**	**11.00 ± 0.3**	9:8 pups	
RMP (mV)				
P1–P5	–60.75 ± 1.5	–57.88 ± 1.6	16:16 cells	0.6435
P10–P12	–60.44 ± 1.8	–59.00 ± 2.1	16:16 cells	
R_IN_ (MW)				
P1–P5	109.2 ± 12.1	128.4 ± 10.5*	16:16 cells	0.0408
P10–P12	87.1 ± 10.3	92.25 ± 11.5	16:16 cells	
I_Hold_ (pA)				
P1–P5	–152.5 ± 25.2	−165.6 ± 21.6	16:16 cells	0.7411
P10–P12	−167.8 ± 30.8	−129.1 ± 31.3	16:16 cells	

All values are mean ± SD. Bold numbers indicate difference between P1–P2 and P10–P12 age groups; * indicates significant difference between eDNE at P1–P5 and control at P10–P12.

### Chronic, eDNE alters repetitive action potential firing in XIIMNs at a critical developmental age

**Figure 2.**
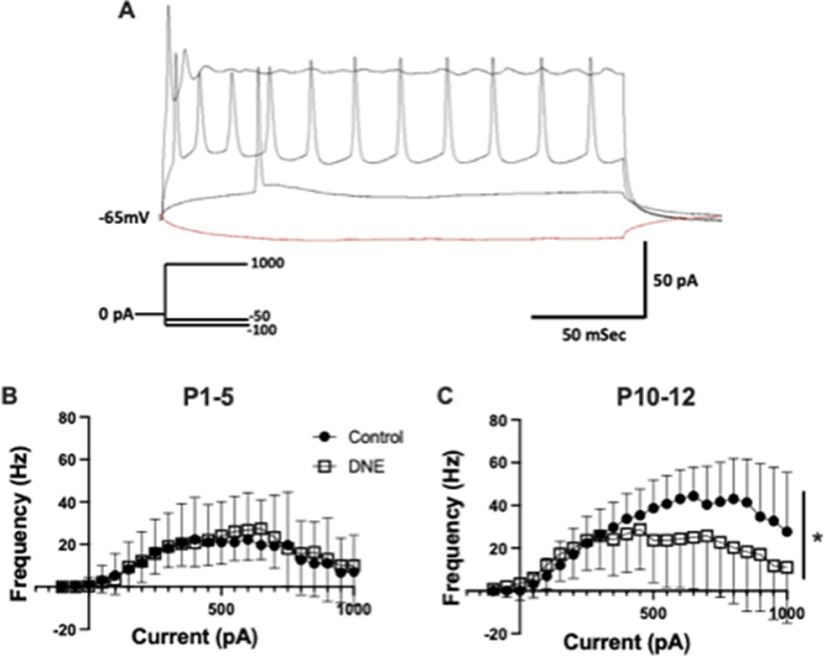
Current-frequency relationship. ***A***, Representative trace showing the response of a P4 control neuron to four of the 23 brief pulses of a current clamp protocol (schematic of protocol shown in inset). Traces, from bottom to top: −100-pA hyperpolarizing current, +150 pA (rheobase) is associated with the onset of action potential firing in this neuron and +800 pA is associated with depolarization block and cessation of action potential firing. ***B***, At P1–P5, there was no difference in the relationship between level of current injection and action potential firing frequency in XIIMNs from control (closed circles) compared with eDNE (open squares) pups. ***C***, At P10–P12, XIIMNs from control pups reached significantly higher average peak firing frequencies compared with XIIMNs from eDNE pups. All data derived from *n* =* *12 control and *n* =* *12 eDNE neurons in each age group. All data presented as mean ± SD; * indicates significant difference between control and eDNE.

To determine whether chronic, eDNE alters excitability or action potential firing rate of XIIMNs we used a current-step protocol. Figure 2*A* is a representative trace of the neuron’s response to current injection in a XIIMN from a control animal at P12, showing the voltage response to four of the 23 current steps applied in the protocol. In this example, the neuron begins firing action potentials at the 100-pA current step, and firing rate rises progressively as a function of the injected current, up to a value of 800 pA, at which time depolarization block occurs and firing rate begins to decline even as injected current continues to increase. The average data showing the relationship between action potential firing rate and current injection in XIIMNs is shown in Figures 2*B*,*C*. At P1–P5, neurons from both eDNE and control pups began firing action potentials at ∼100-pA injected current, and firing frequency peaked at ∼30 Hz when injected current ranged between ∼400–800 pA. In this age group, action potential firing properties were not different between control and eDNE (*F* = 0.1986, *p* = 0.6602), nor was the current step corresponding with initial action potential firing (control: 250 ± 240.3 pA, eDNE: 162.5 ± 90.77 pA) or depolarization block (control: 758.3 ± 62.11 pA, eDNE: 787.5 ± 60.65 pA). By P10–P12, although the current step associated with initial action potential firing was not different between control and eDNE (control: 145.8 ± 101.0 pA, eDNE: 116.7 ± 126.7 pA), neurons from control pups reached higher peak action potential firing frequencies (∼45 Hz) compared with neurons from eDNE pups (∼30 Hz; *F* = 4.805, *p* = 0.0392), and neurons from eDNE pups reached depolarization block at significantly lower levels of current (658.3 ± 84.35 pA) compared with neurons from control pups (945.8 ± 28.51 pA; *p* = 0.0191). Data obtained from *n* = 12 control and eDNE neurons in each age group).

### Chronic, eDNE alters voltage-sensitive potassium currents in XIIMNs at a critical developmental age

**Figure 3.**
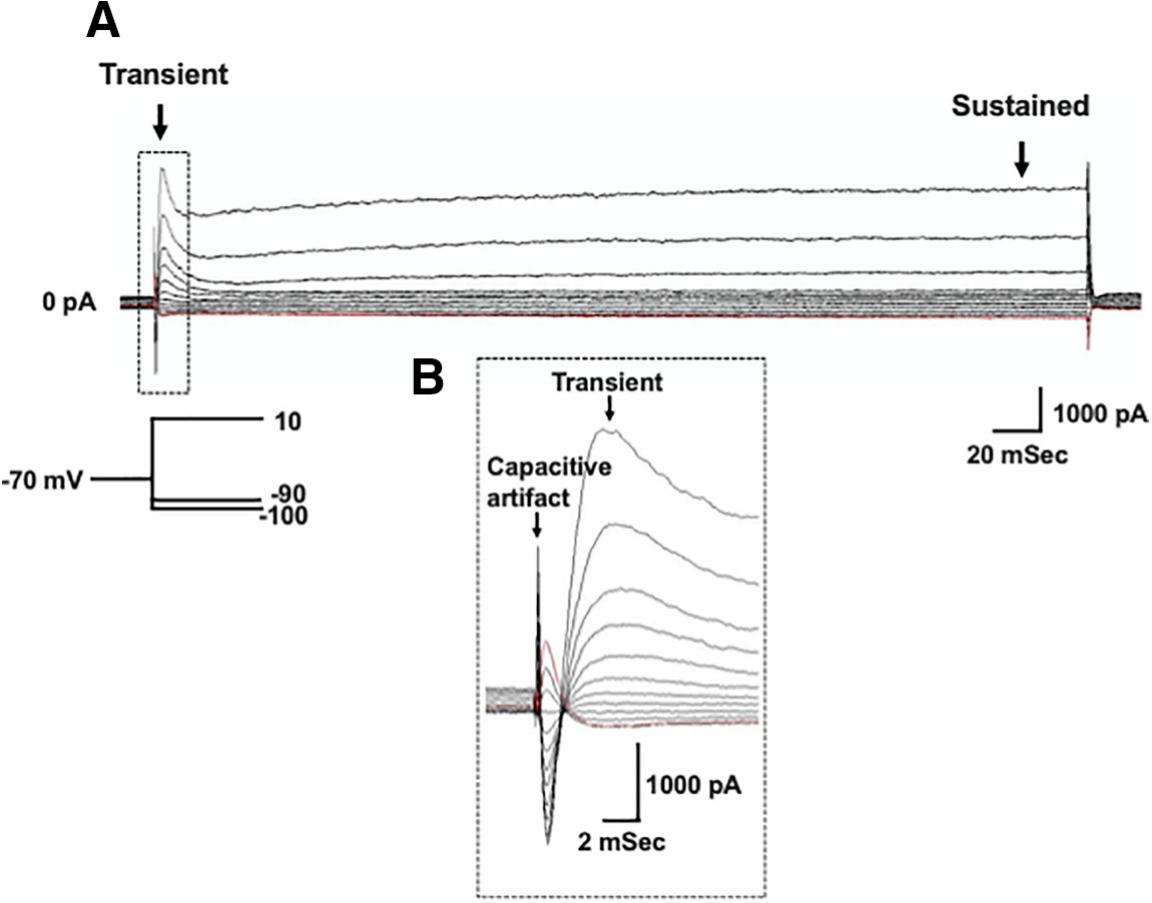
Whole-cell potassium currents. ***A***, Representative recording demonstrating the whole-cell potassium current recorded in the presence of TTX. From a holding potential of −70 mV, voltage was stepped to –100 mV, followed by depolarizing steps in 10-mV increments to a final voltage of +10 mV (schematic of voltage step protocol shown in *inset*). Arrows indicate where measurements were made for the transient (left) and sustained (right) components of the current. ***B.*** An expanded view of the portion of the trace outlined by the dotted box in ***A***, showing the capacitive artifact, followed by the rapidly activating and inactivating, transient current component.

**Figure 4.**
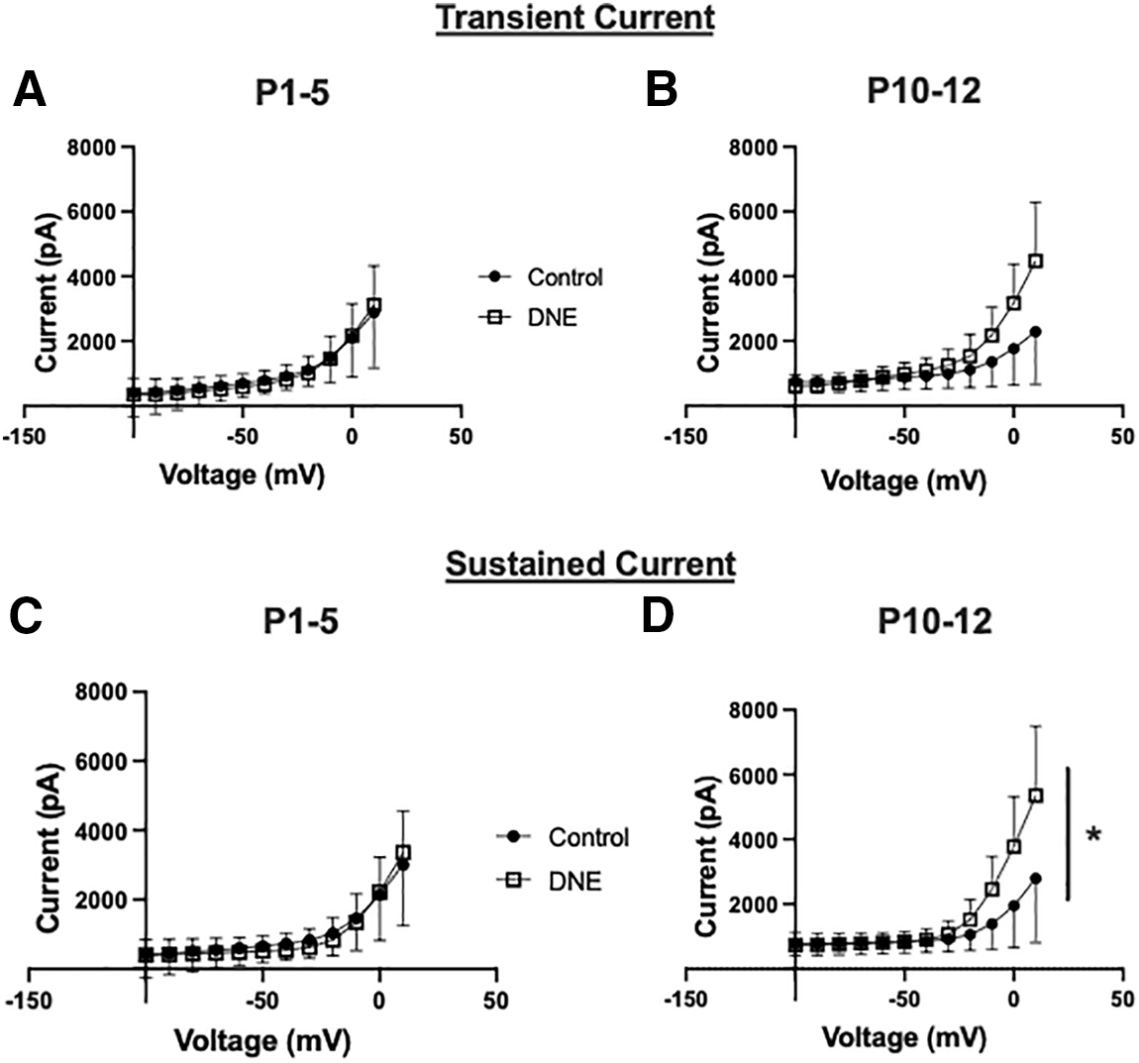
Summary data of whole-cell potassium currents. There was no difference in the transient current component between XIIMNs from control (closed circles) and eDNE (open squares) pups at either P1–P5 (***A***) or P10–P12 (***B***). ***C***, At P1–P5, the sustained current component was not different between XIIMNs from control and eDNE pups, however, by P10–P12 (***D***), XIIMNs from eDNE pups exhibited larger currents compared with those from control pups. Data derived from *n *=* *12 control neurons and *n *=* *12 eDNE neurons in each age group. All data presented as mean ± SD. *, indicates significant difference between control and eDNE.

**Figure 5.**
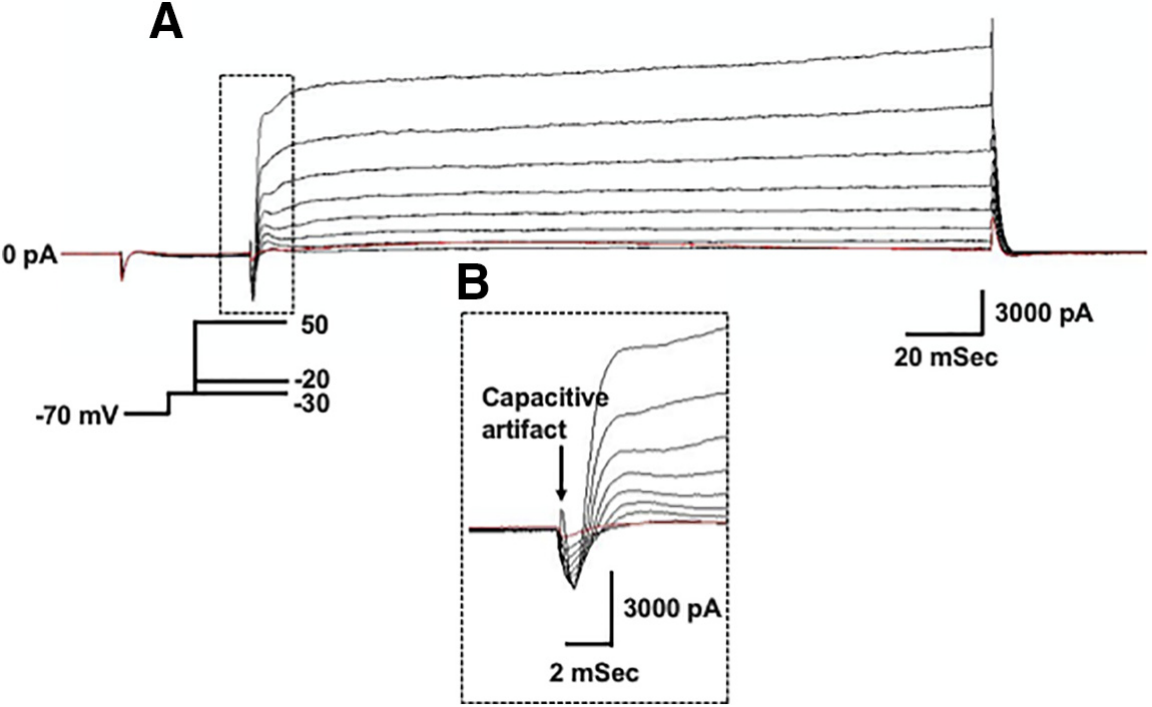
Prepulse protocol abolishes A-type potassium currents. ***A***, Representative recording demonstrating the prepulse protocol recorded in the presence of TTX. From a holding potential of −70 mV, the membrane potential was stepped first to −30 mV, inactivating the A-type potassium current, followed by depolarizing voltage steps applied in 10-mV increments to a final potential of +50 mV (schematic of voltage step protocol shown in inset). ***B***, An expanded view of the portion of the ***A*** trace outlined by the dotted box showing the capacitive artifact and initial outward current. Note that in the fast activating and inactivating current component that we see in the whole-cell trace (Fig. 3) is abolished.

Voltage gated potassium currents are important regulators of neuron excitability and repetitive firing. Therefore, we wanted to test whether the decrease in action potential firing rate observed in XIIMNs from P10–P12 eDNE pups is associated with altered potassium currents. After bath application of TTX, we first used a voltage-step protocol to record whole-cell potassium currents where, from a −70-mV holding potential, we varied the membrane potential stepwise from −100 to +10 mV. Figure 3*A* shows an example trace recorded in a XIIMN from a P10 control pup. As seen in this trace, shortly after the capacitive artifact, we observed a rapidly activating and inactivating transient outward current, which is shown in an expanded view within the dotted rectangle (Fig. 3*B*). The downward arrow indicates where we measured the amplitude of this transient current. As seen in Figure 4*A*, in neurons from P1–P5 pups, there was no difference in the transient current amplitude between control and eDNE (*F* = 0.04283, *p* = 0.8379). By P10–P12, there was a trend toward larger transient currents at the most depolarized voltage steps in neurons from eDNE pups compared with control (Fig. 4*B*), although this was not statistically significant (*F* = 3.502, *p* = 0.0746). In a separate experiment, we sought to determine whether the rapidly activating and inactivating, A-type potassium current contributed to the transient current component of the whole-cell current. To do this, we conducted a protocol that began with a prepulse to −30 mV (Fig. 5*A*), which inactivates the A-type current, followed by the same step protocol described for whole-cell currents above. As shown in Figure 5, the prepulse to −30 mV nearly abolishes the transient potassium current (expanded view shown in Fig. 5*B*), indicating that the transient component of the whole-cell current in XIIMNs is mediated by the A-type potassium current.

**Figure 6.**
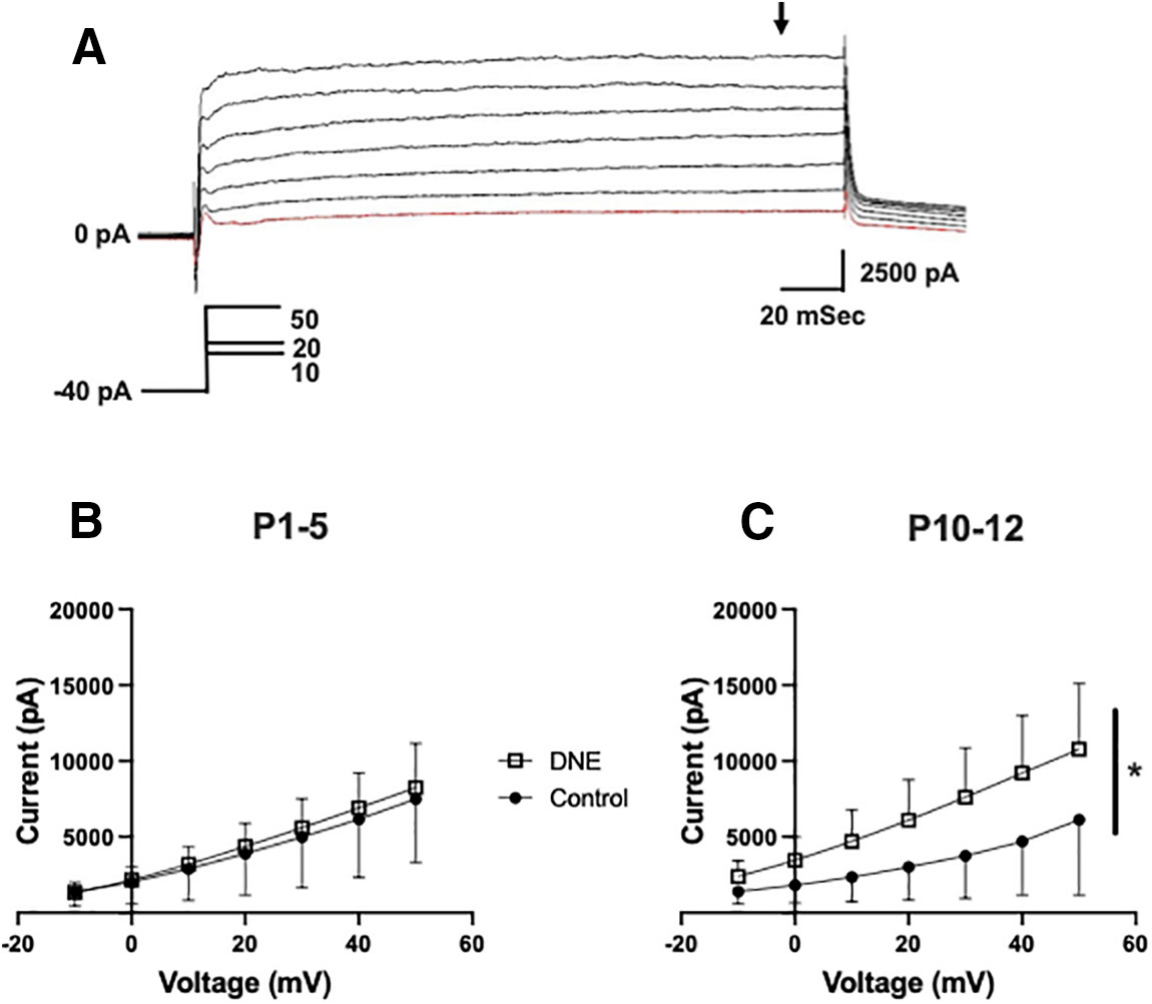
Delayed-rectifier potassium currents. ***A***, Representative recording demonstrating the isolated delayed rectifier potassium current recorded in the presence of TTX. From a holding potential of −40 mV, which inactivates the transient A-type potassium channels, depolarizing voltage steps were applied in 10-mV increments to a final potential of +40 mV (schematic of voltage step protocol show in inset). ***B***, At P1–P5, the sustained current component was not different in XIIMNs from control (closed circles) and eDNE (open squares) pups; however, by P10–P12 (***C***), XIIMNs from eDNE pups exhibited larger currents compared with those from control pups. Data derived from *n *=* *12 control neurons and *n *=* *12 eDNE neurons in each age group. All data presented as mean ± SD. Arrow in ***A*** indicates where current was measured; * indicates significant difference between control and eDNE.

We also measured the sustained outward current in the whole-cell current trace (Fig. 3*A*), with the measurement taken near the end of the current step, as indicated by the rightmost arrow. As shown in Figure 4*C*, at P1–P5, the amplitudes of the sustained current component were virtually identical in neurons from control and eDNE pups (*F* = 0.06757, *p* = 0.7973). However, by P10–P12, the sustained potassium currents were significantly larger in neurons from eDNE pups compared with control pups (Fig. 4*D*; *F* = 4.306, *p* = 0.0499). Next, we used a voltage step protocol that isolates the more slowly activating voltage-dependent, delayed rectifier current. An example trace recorded in a XIIMN from an eDNE pup is shown in Figure 6*A*. Because the delayed rectifier current does not inactivate, while the A-type potassium current does, we used a protocol that starts at a holding potential of −40 mV. Setting the holding current to −40 mV inactivates the A-type potassium current, but the delayed rectifier current remains. As shown in Figure 6*B*, there was no difference in delayed rectifier currents in neurons from control and eDNE pups at P1–P5 (*F* = 0.1650, *p* = 0.6885). However, by P10–P12 (Fig. 6*C*), neurons from eDNE pups had larger delayed rectifier currents compared with neurons from control pups (*F* = 7.319, *p* = 0.0145; data obtained from *n* = 12 control and eDNE neurons in each age group).

### Chronic, eDNE alters nAChR desensitization and recovery in XIIMNs from neonatal rats

**Figure 7.**
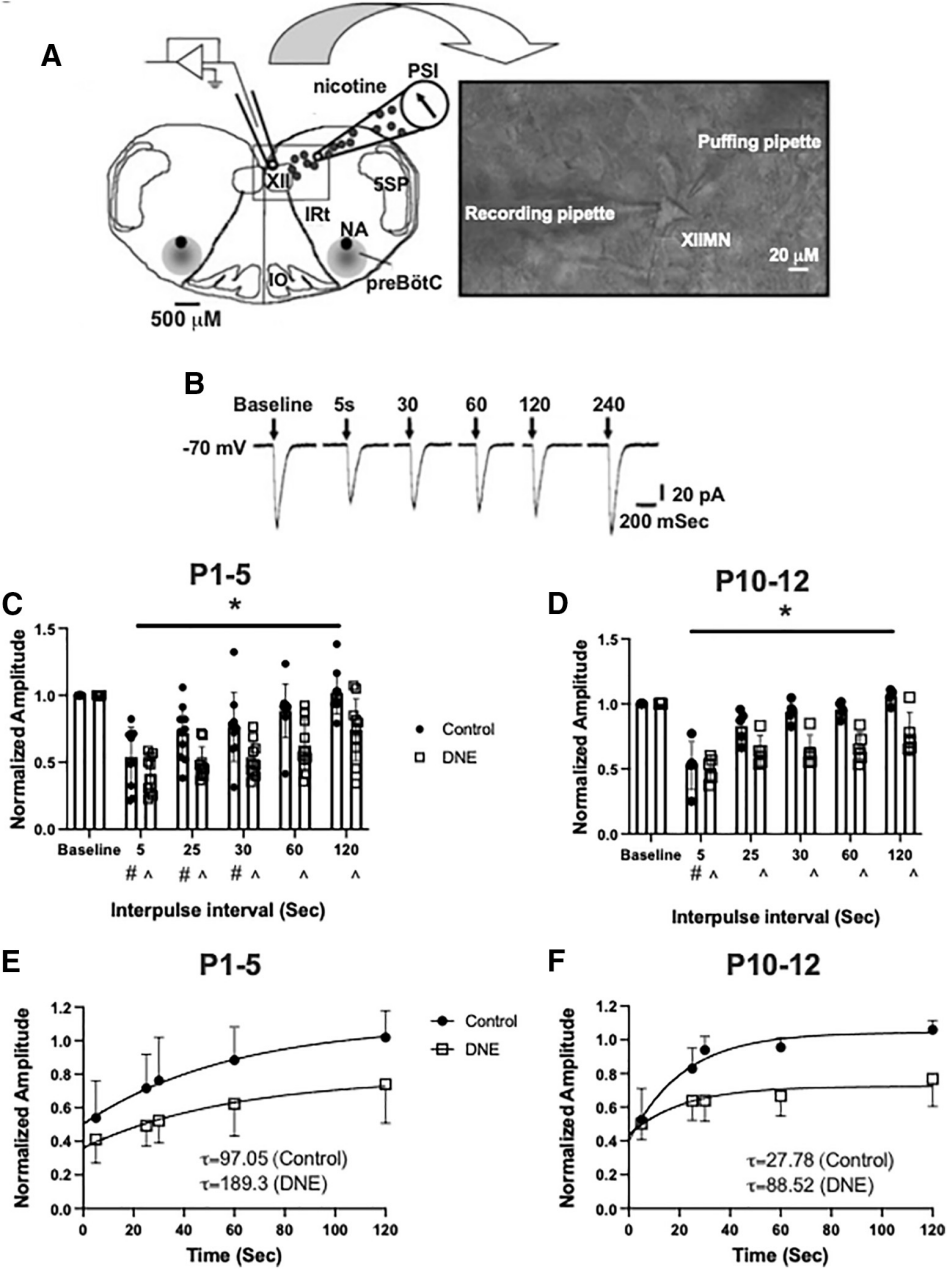
nAChR desensitization and recovery. ***A***, A representation of the experimental set-up showing the rostral surface of the medullary slice preparation with landmarks including the XII motor nucleus, trigeminal nucleus (5SP), preBötzinger complex, nucleus ambiguus (NA), inferior olivary nucleus (IO), and the intermediate reticular formation (IRt). Inset shows a high-resolution image (40×) of an isolated XII MN with a recording pipette and a “puffing” pipette. ***B***, Examples of inward currents produced by nicotine pressure pulses (2 mm; 30 ms) in a P4 conditioning neuron during a 4-min pressure-pulse protocol. Pressure pulses occurred at successively longer intervals following a conditioning pulse at time 0. At both P1–P5 (***C***) and P10–P12 (***D***), eDNE (open squares) causes a delay in the recovery of nAChR-mediated peak currents compared with control (closed circles). ***E***, ***F***, Quantification of nAChR recovery using a single exponential fit to the average data shown in ***C***, ***D***. eDNE slows recovery at both age groups as indicated by time constants (τ), shown in insets. Data derived from *n *=* *12 control neurons and *n *=* *12 eDNE neurons in each age group. All data presented as mean ± SD; in ***C***, ***D*** # indicates significant difference from baseline within control group; ^ indicates significant difference from baseline within eDNE group. ***A*** reproduced with permission from Pilarski et al. (2012).

Native rat nAChRs are known to desensitize in a time-dependent manner(Quitadamo et al., 2005). Here, we quantified nAChR desensitization and recovery by administering a pressure pulse protocol that included an initial conditioning pulse of nicotine, followed by subsequent pulses at increasing intervals (Fig. 7*B*). Previous studies have observed that as the duration of the interpulse interval increases, the peak current amplitude progressively recovers (Paradiso and Steinbach, 2003), and that the rate of recovery from desensitization is blunted by chronic perinatal nicotine exposure (Pilarski et al., 2012). At P1–P5, there was no difference in the overall magnitude of nAChR desensitization, with the second pulse resulting in current amplitudes of 53.9 ± 0.071% and 41.1 ± 0.045% of the initial current in neurons from control and eDNE pups, respectively (Fig. 7*C*). Recovery from desensitization, however, was blunted in neurons from eDNE pups compared with control. In neurons from control pups, current amplitudes were not different from baseline with the fourth or fifth nicotine pulse, however, in neurons from eDNE animals, current amplitudes were decreased compared with baseline through all subsequent nicotine pulses (time: *F* = 52.39, *p* < 0.0001; treatment: *F* = 9.498, *p* = 0.006). The rate of recovery was estimated by computing the time constant for recovery, which averaged 97.05 s for control versus 189.3 s for eDNE (Fig. 7*E*). At P10–P12, the second nicotine pulse resulted in a similar decrease in current amplitude between the two treatment groups (control: 52.5 ± 0.083% baseline; eDNE: 50.3 ± 0.042% baseline). In neurons from control pups, only the second pulse was lower than baseline, however in eDNE neurons, the amplitude of all subsequent pulses was less than baseline (time: *F* = 27.01, *p* < 0.0001; treatment: *F* = 26.52, *p* = 0.0009; Fig. 7*D*). The time constant for recovery averaged 27. 8 s for control versus 88.5 s for eDNE (P1–P5: data obtained from *n* = 10 control and eDNE neurons; P10–P12: data obtained from *n* = 5 control and eDNE neurons; Fig. 7*F*).

### Chronic, eDNE is associated with blunted tongue muscle EMG response to nasal occlusion at a critical developmental age

**Figure 8.**
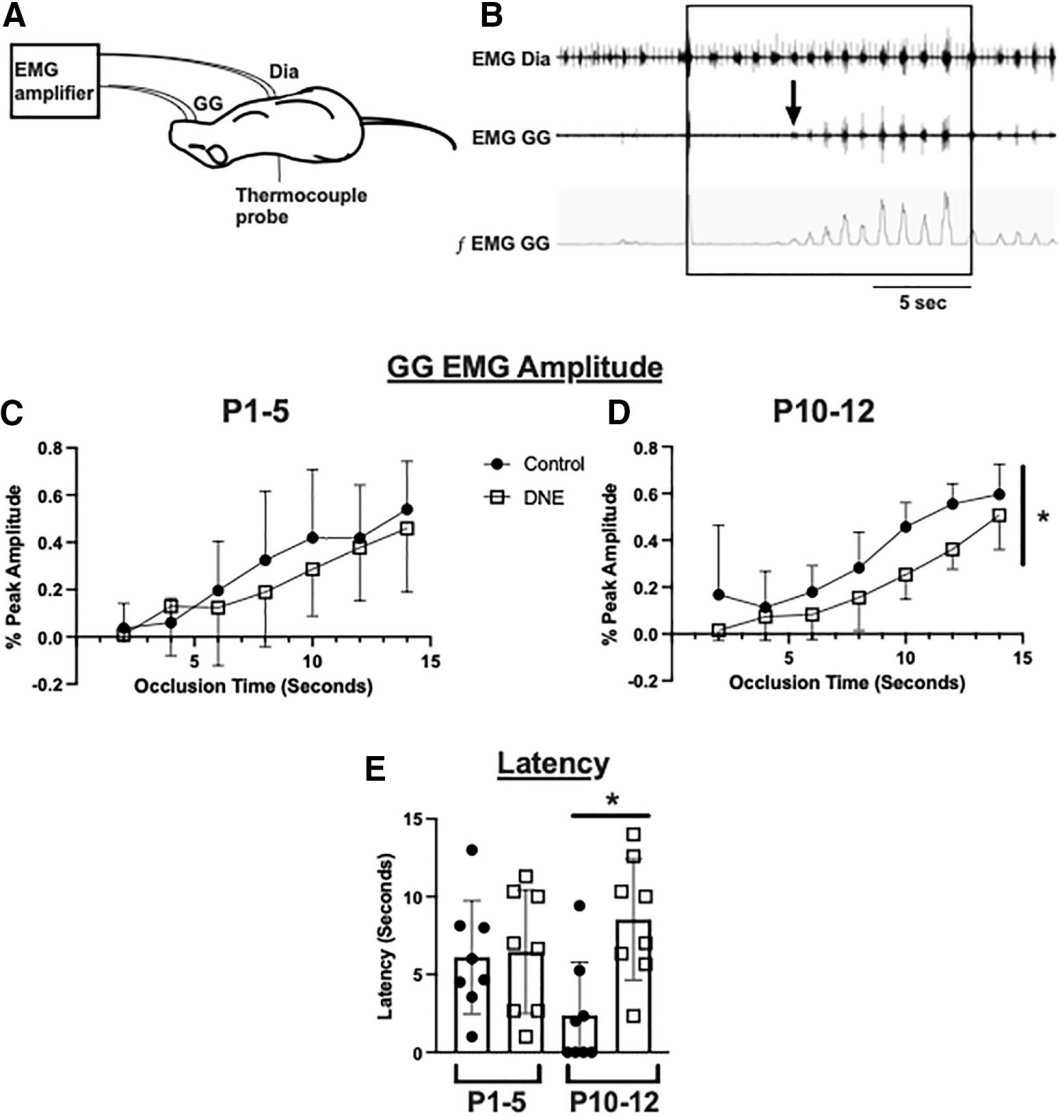
*In vivo* experimental preparation. ***A***, A schematic of the *in vivo* experimental preparation which includes concurrent diaphragm (DIA) and GG EMG recordings in lightly anesthetized rats. For details, see Materials and Methods. High-frequency spikes in the diaphragm EMG are EKG artifacts. ***B***, Example trace showing diaphragm and GG EMG and the rectified and integrated GG EMG. After uninterrupted baseline recordings, a 15-s nasal occlusion was administered (indicated by square). The distance between the onset of nasal occlusion and the first discernable GG muscle EMG burst (arrow) is defined as onset latency. ***C***, ***D***, EMG amplitude was normalized as a percentage of the largest burst recorded during nasal occlusion (see Materials and Methods). At P1–P5, GG EMG amplitude during nasal occlusion was not different between control (closed circles) and eDNE (open squares) pups (***C***); however, by P10–P12, eDNE animals had a significantly blunted amplitude response compared with control animals (***D***). At P1–P5, onset latency of GG EMG bursts was not different between control and eDNE pups (***E***). However, by P10–P12, onset latency of GG EMG bursts was significantly longer in eDNE pups compared with control pups. Data derived from *n *=* *8 control pups and *n *=* *8 eDNE pups in each age group. All data presented as mean ± SD; * indicates significant difference between control and eDNE.

The results from our electrophysiology experiments show that eDNE results in altered XIIMN firing properties at P10–P12, and altered function of nAChRs on XIIMNs in both age groups. To determine whether these cellular changes correlated with altered tongue muscle function *in vivo*, we did EMG recordings of the GG muscle of the tongue (Fig. 8*A*), which is innervated by XIIMNs and plays an important role in maintaining upper airway patency (Remmers et al., 1978). We challenged the system with a 15-s nasal occlusion, initiated during the expiratory period, which results in a strong increase in drive to the respiratory muscles during breathing. As shown in Figure 8*B*, nasal occlusion is associated with a monotonic increase in the EMG activity of both the diaphragm (top trace) and GG (bottom two traces). At P1–P5, GG activity during occlusion was not different between control and eDNE pups (time: *F* = 18.67, *p* < 0.0001; treatment: *F* = 0.5923, *p* = 0.4543; Fig. 8*C*). However, at P10–P12, GG EMG activity during nasal occlusion was significantly blunted in the eDNE animals (time: *F* = 38.38, *p* < 0.0001; treatment: *F* = 10.17 *p* = 0.0066; Fig. 8*D*). In addition to the reduced excitation of XIIMNs in response to the stress of nasal occlusion, the onset latency was longer in eDNE animals at P10–P12 (*F* = 3.783, *p* = 0.0214), whereas it was not different between treatment groups at P1–P5 Fig. 8*E*; data obtained from *n* = 8 control and *n* = 8 eDNE pups in both age groups).

## Discussion

We studied the effects of chronic, eDNE on nAChR function and XIIMN intrinsic properties *in vitro* and tongue muscle function *in vivo* in neonatal rats at two developmental stages. At P1–P5, eDNE animals showed enhanced desensitization and delayed recovery of nAChRs after brief pressure applications of nicotine, however no abnormalities in XIIMN intrinsic properties were observed and eDNE pups exhibited a normal tongue muscle EMG response to nasal occlusion. By P10–P12, which encompasses the days just before and during a critical period in respiratory system development, altered nAChR function was still observed, and XIIMNs also exhibited altered firing properties and enhanced potassium currents in neurons from eDNE pups. At this age, these changes were accompanied by a blunted tongue muscle EMG response to nasal occlusion, and an increased EMG response latency in eDNE pups compared with controls. This indicates that altered intrinsic properties of XIIMNs is an important mechanism behind this effect, which is not produced by changes in nAChR function alone. The significance of these results is discussed below.

### Do changes in nAChR desensitization kinetics explain the effects of eDNE on XIIMNs?

nAChRs modulate neurotransmission and there is a large body of evidence that shows nAChR activation can also activate several signal transduction pathways, leading to persistent cellular alterations in the brain (Barik and Wonnacott, 2009). nAChR signaling plays a crucial role in brain development, and in the development of rhythmic motor circuits, including circuits that control the muscles of breathing (Greer et al., 2006). nAChRs normally undergo desensitization, a reversible reduction in response during sustained agonist application. Although the role of nAChR desensitization in normal cholinergic transmission is unclear, it is thought to shape synaptic plasticity and protect cells from uncontrolled excitation (Giniatullin et al., 2005). Given the importance of the nicotinic cholinergic system in brain ontogenesis, it is evident that nicotine could have severe consequences on brain development and function. Like previous studies using continuous exposure with osmotic minipumps (Pilarski et al., 2012), we show here that eDNE results in delayed recovery of nAChRs on XIIMNs in rats aged P1–P5 and P10–P12. This is consistent with studies showing that with the continuous presence of nicotine, nAChRs slowly reach a long-lasting inactive or desensitized state which is thought to be a trigger for upregulation of membrane surface nAChRs, which in turn also desensitize (Fenster et al., 1999a; Quick and Lester, 2002). Therefore, although the number of nAChRs is often increased with chronic nicotine exposure, in many regions of the brain a larger fraction are unavailable to respond to agonists (Gentry and Lukas, 2002). Protein kinase C-dependent phosphorylation of α−4 subunit containing nAChRs slows recovery from the desensitized state, and this mechanism has been hypothesized to lead to the long-lasting inactivation of α−4:β−2 containing nAChRs often observed in chronic nicotine exposure models (Fenster et al., 1999b). Whether recovery from this state simply requires adequate time, or if new receptors are necessary to regain function, is not known (Boyd, 1987). Nevertheless, long-lasting or permanent desensitized states likely promote synaptic plasticity that results in the cellular changes associated with altered structure and synaptic function of neonatal neurons that govern many important behaviors including the control of breathing (Yu et al., 2010; Lester, 2011; Jaiswal et al., 2013, 2016; Garcia-Bereguiain et al., 2016; Wollman et al., 2018b,c; Buls Wollman et al., 2019).

In previous work, desensitization was accompanied by various changes to XIIMNs, including a decrease in XIIMN size, a less complex dendritic arbor (Pilarski et al., 2011; Powell et al., 2015), and increased input resistance and hyperexcitability (Pilarski et al., 2011). Proper functioning of neuronal networks relies on both the appropriate balance of excitatory and inhibitory influences on a neuron, as well as an appropriate postsynaptic response. Perturbation of neuronal signaling leads to changes in synaptic efficacy which must be accompanied by homeostatic mechanisms that prevent hyperactivity or hypoactivity (Turrigiano and Nelson, 2004). Therefore, a proposed working hypothesis is that nAChR desensitization, together with other fundamental developmental changes in XIIMNs observed with DNE, results in increased neuron excitability, which in turn triggers compensatory plasticity mechanisms directed at restoring excitability back toward normal. Examples of compensatory mechanisms that have been observed include enhanced potassium conductances (Cholanian et al., 2017), reduced excitatory synaptic inputs and glutamate receptor expression (Jaiswal et al., 2013), and enhanced postsynaptic inhibition of XIIMNs (Jaiswal et al., 2013; Wollman et al., 2018a,c).

### eDNE alters XIIMN intrinsic properties at P10–P12 but not at P1–P5

In the present study, as in studies using continuous nicotine exposure with the osmotic minipump, we find that chronic, eDNE is associated with enhanced desensitization and delayed recovery of nAChRs at P1–P5, and that this effect persists to the P10–P12 age group. In contrast, we did not observe changes in input resistance, firing threshold, or the initial slope (gain) of the current-frequency relationship with eDNE at either age. However, compared with control neurons, XIIMNs from eDNE animals showed lower peak firing rates in response to current injection, but only in the P10–P12 age group. The reduction in XIIMN peak firing rates in nicotine exposed pups at this age is accompanied by a significantly greater delayed rectifier potassium conductance. This conductance is critical for producing the afterhyperpolarization that permits sufficient time for recovery of voltage gated sodium channels from inactivation. Some neurons, including CA1 pyramidal neurons, have a reserve of voltage-gated sodium channels that appears to allow for continued action potential firing even when a significant portion of the total number of channels are inactivated from previous activity (Madeja, 2000). Multiple studies in motor neurons, including XIIMNs, show that maximal firing rates increase over early postnatal development (Viana et al., 1995; Carlin et al., 2008). In spinal motor neurons, this observation is attributed to an increase in sodium channel density (Gao and Ziskind-Conhaim, 1998; García et al., 1998), and a more pronounced after-hyperpolarization during repetitive spiking (Fulton and Walton, 1986). Based on this, we consider the possibility that eDNE alters the normal expression of voltage-gated sodium channels, resulting in altered firing properties of XIIMNs; the increase in delayed rectifier potassium currents seen in the present study may be an attempt, albeit unsuccessful, to restore firing rates back toward normal.

### eDNE alters the tongue muscle EMG response to nasal occlusion

Inadvertent airway obstruction, such as when an infant’s head gets covered by bedding, is accompanied by an increase in blood CO_2_ and a decrease in O_2_, leading to an increase in the release of several neurotransmitters, including ACh, the endogenous ligand for nAChRs. The appropriate changes in excitatory synaptic inputs, along with the postsynaptic response of the motor neurons, are both critical to producing an effective respiratory motor response to chemoreceptor stimulation (Metz, 1966). This response includes increased drive to the respiratory muscles, including the muscles of the tongue, which contribute importantly to the maintenance of airway patency during inspiration. Critical to this work, the main respiratory phenotype observed in nicotine-exposed human infants is an increased incidence of obstructive apneas, which is thought to be a pathophysiological precursor to SIDS (Newborn, 2003). In previous work, we found that DNE using the osmotic minipump model was associated with a delayed and blunted GG muscle motor response to nasal airway occlusion in pups at ages P3–P7. Here, we found that eDNE causes similar changes in respiratory-related tongue muscle function, however only at ages P10–P12. It is not clear whether the blunted tongue muscle response to chemoreceptor stimulation is because of concomitant changes in intrinsic XIIMN properties, reduced excitatory synaptic input (Wollman et al., 2019), increased inhibitory synaptic inputs (Wollman et al., 2018b,c) or a DNE-mediated blunting of peripheral and central chemoreceptor reflexes (Hafström et al., 2005; Campos et al., 2009). However, based on these results, changes in XIIMN intrinsic properties are at least one important mechanism behind this effect, as it cannot be explained by altered nAChR function alone.

### Continuous versus episodic nicotine delivery

The present findings are like what has been shown using the osmotic minipump model of DNE, with some key differences. The most substantial difference observed is that other than altered nAChR function, the other effects of eDNE (described above) are not present in the first week of life. Here, we found that nicotine delivery through drinking water resulted in plasma cotinine concentrations in the pups that were like what we have seen previously using the osmotic pump at these ages. While plasma cotinine concentrations are widely used as an indicator of nicotine exposure, pharmacokinetic studies of nicotine and cotinine show that cotinine blood levels are not a good measure of brain nicotine concentrations (Ghosheh et al., 2001). Thus, differences between our current model and the minipump exposure models might be explained simply by a nicotine dose effect, i.e., intermittent versus continuous DNE exposure models may result in significantly different brain nicotine concentrations. Another possibility is that the pattern of nicotine exposure determines the effects of nicotine. Such pattern dependence can be observed in respiratory motor neurons in response to pharmacologic activation of serotonin receptors or to systemic hypoxia, where intermittent exposure triggers facilitation of respiratory motor output but sustained exposure does not (Baker and Mitchell, 2000; Bocchiaro and Feldman, 2004; Lovett-Barr et al., 2006). Differences in the effects of intermittent versus sustained stimulation have been found in multiple other neural systems and animal species as well, including sensory adaptation in *Aplysia* (Mauelshagen et al., 1998), olfactory conditioning in *Drosophila* (Beck et al., 2000), and multiple forms of hippocampal plasticity in mice (Kogan et al., 1997).

### The critical period in respiratory-system development and implications for SIDS

One of the most influential hypotheses put forward to explain SIDS proposes that the coincidence of three factors increases the risk of death: a “vulnerable” infant, an external stressor, and a critical developmental period (Filiano and Kinney, 1994). Near the end of the second week of life in rats, there is a transient period of excitatory/inhibitory imbalance that is thought to be a critical time window for shaping and refinement of the respiratory control network (Carroll, 2003). This critical period appears to be genetically predetermined, as it can be delayed or prolonged by external stimuli, but not eliminated.

The present study presents a significant advancement in our knowledge of the role of DNE in the pathogenesis of respiratory control abnormalities that may underlie SIDS. Although continuous nicotine delivery via osmotic pumps models the nicotine patch, the episodic exposure model used here more realistically mimics the delivery of nicotine by cigarettes, nicotine gum, and electronic delivery devices. In addition, in much of the previous work, the effects of DNE were studied in neonatal rats within the first week of life, which roughly correlates to the third trimester in fetal development in humans (Agoston, 2017). In the present study, we also used rats at later ages, P10–P12, which correlates with the first few days of neonatal life in humans, closer to the age where some SIDS deaths occur. Finally, we found that abnormalities in nAChR function and XIIMN firing properties identified at P10–P12 are associated with altered respiratory-related tongue muscle control *in vivo,* which is a novel finding that elucidates important mechanisms behind how DNE leads to a behavioral vulnerability at a critical period of respiratory system development, a phenotype that nicely models key aspects of the pathogenesis of SIDS.

## Synthesis

Reviewing Editor: Silvia Pagliardini, University of Alberta Decisions are customarily a result of the Reviewing Editor and the peer reviewers coming together and discussing their recommendations until a consensus is reached. When revisions are invited, a fact-based synthesis statement explaining their decision and outlining what is needed to prepare a revision will be listed below. The following reviewer(s) agreed to reveal their identity: Gordon Mitchell. Note: If this manuscript was transferred from JNeurosci and a decision was made to accept the manuscript without peer review, a brief statement to this effect will instead be what is listed below.

The study investigates the effect of chronic episodic perinatal exposure of nicotine on nAChR function, the intrinsic properties of hypoglossal motoneurons in in vitro medullary slice preparation and the activity of the tongue muscle in vivo.

Several studies have demonstrated profound differences in the respiratory motor system in response to continuous versus episodic stimulation. This is the first study to show that the pattern of nicotine exposure is a determinant of upper airway motor neuron responses. Specifically, episodic (eg. vaping, nicotine gum) versus continuous (eg. nicotine patch) nicotine exposure have different impact in a developmentally specific context. The results here may/may not be relevant to SIDS, which is strikingly associated with maternal smoking.

The data showing differences in K+ currents and nAChR desensitization in hypoglossal motoneurons are important for the field and may provide insight into the higher prevalence of respiratory pathology in the children of smokers.

This is a manuscript that was transferred from JNeuroscience and underwent revisions to address previous reviewer comments. As a result, the revised manuscript has been significantly improved, and the changes have strengthened the manuscript and data presentation.

The previous reviews pointed out that these data are largely overlapping with previous studies investigating neurophysiological changes following prenatal nicotine exposure, which is still a concern. Having said that, the use of episodic treatments rather than continuous exposure is important and the results are quite interesting in their similarity to previous findings.

The authors dismissed requests for a more systematic comparison of these data with previous findings with continuous nicotine exposure, and this comparison is important, even if results are largely similar.

However, there are minor concerns the authors may wish to address, relating to responses to the prior review. In specific, the authors should evaluate the following statements and potentially revise to make them more accurate:

1. The change in nAChR desensitization with intermittent developmental nicotine exposure is quite interesting, and it would be great if the authors could provide more mechanistic insights. At a minimum they should consider describing possible underlying mechanisms in the Discussion, ie phosphorylation state.

2.There are important differences in the behavioral effects of nicotine in males and females. It is problematic that the authors have not included information about animal gender in their results. Response magnitudes should be compared between the sexes.

3. Page 19, end of section on continuous versus episodic: the comment is not accurate: “Another possible.....” In specific, the continuous versus sustained hypoxia discussed relates to competing serotonergic versus adenosinergic mechanisms and is not specifically related to gene expression in this time frame. The passage makes it sound like serotonin is the only issue and that regulation of gene expression is the outcome.

4. In the final paragraph of the discussion, the statement “closer to a time when most SIDS deaths occur” seems slightly exaggerated (although true). Most SIDS deaths in babies occur between 1 and 12 months of life, a period delayed from the time frame the authors are discussing.
